# Informant characteristics are associated with the Clinical Dementia Rating Sum of Boxes scores in the Alzheimer’s Disease patients participating in the National Alzheimer’s Coordinating Center Uniform Data Set

**DOI:** 10.21203/rs.3.rs-3982448/v1

**Published:** 2024-03-15

**Authors:** Juan-Camilo Vargas-Gonzalez, Antonella Santuccione Chadha, Laura Castro-Aldrete, Maria Teresa Ferretti, Carmela Tartaglia

**Affiliations:** University Health Network; Women’s Brain Project; Women’s Brain Project; Center for Alzheimer Research, Karolinska Institut; University of Toronto

## Abstract

**Background:**

The Clinical Dementia Rating^®^ Sum of Boxes (CDR^®^-SB) is used to stage dementia severity; it is one of the most common outcome measurements in Alzheimer’s Disease (AD) research and clinical trials. The CDR^®^-SB requires an informant to provide input to stage a patient’s dementia severity. The effect of the informant’s characteristics on the CDR^®^-SB is unknown. We aimed to evaluate the effect of the informant’s sex, relationship to the patient, and frequency of contact on the CDR^®^-SB scores in patients with Alzheimer’s Disease with mild cognitive impairment or dementia included in the National Alzheimer’s Coordinating Center Uniform Data Set (NACC-UDS).

**Methods:**

We included all participants from the NACC-UDS that had AD as diagnosis, and information about the Mini-Mental State Examination or Montreal Cognitive Assessment scores, informant sex, relationship to patient and frequency of contact; we also analyzed the possible interaction between these characteristics on the CDR^®^-SB as the outcome. We performed a multilevel linear regression analysis.

**Results:**

We included data from 20636 participants, totalling 47727 visits. Patients’ age was 74.0 ± 9.4 years and 54.1% were females. Informant characteristics were mean age of 66.2 ± 13.2 years, 69.1% were females, and the relationship to patients was 60.5% spouse or partner, 26.7% children and 12.8% other relation. The CDR^®^-SB scores were 0.20 higher (CI 95%: 0.11 to 0.29) when the informant was female. When comparing to informant’s relationship with the baseline being spouse or partner, the CDR^®^-SB was 0.39 higher (CI 95%: 0.25 to 0.53) when the informant was the patient’s child and 0.18 lower (CI 95%: −0.35 to −0.01) if relationship was other. Regarding the frequency of contact, CDR^®^-SB scores were 0.38 higher (CI95%: 0.28 to 0.47) when contact was at least once a week, 0.65 higher (CI95%: 0.52 to 0.78) when contact was daily, and 0.57 higher (CI95%: 0.46 to 0.69) when informant was living with the patient, baseline was a frequency of less than once per week. Finally, the interaction between informant relationships other and female patients showed a 0.24 higher CDR^®^-SB score (CI95%: 0.03 to 0.46).

**Conclusions:**

We found that the CDR^®^-SB scores are significantly modified by informant characteristics and frequency of contact in the NACC-UDS patients with AD diagnosis. These findings hold clinical significance as informant characteristics ideally should not impact the staging of AD patients, and any such effects could introduce bias into clinical evaluations in clinical trials. Future research endeavours should investigate strategies to address and mitigate the influence of these confounding variables.

## Introduction

Assessing disease severity in dementia is a multi-factorial endeavour that involves a comprehensive evaluation of cognitive, functional, behavioural, and psychological aspects. Various tools and methods are used by healthcare professionals to gain insights into the individual’s condition. The Clinical Dementia Rating^®^ Dementia Staging Instrument (CDR^®^) is a widely used tool to assess the severity of dementia. It provides a global rating of dementia severity based on structured interviews and evaluations. The CDR^®^ categorizes individuals into different stages, ranging from no impairment (CDR^®^ 0) to severe impairment (CDR^®^ 3). Disease staging allows discrete category classification of disease evolution wherein categories are clinically detectable and reflect disease severity in terms of meaningful risk of disability, impairment or death (1). Staging can be used to adequately assess the severity of disease, to help determine the most appropriate treatment, and even to inform prognosis(2). The CDR^®^ was developed in the 1980s as a tool to standardize staging in Alzheimer’s Disease (AD). It has since then been validated and adopted worldwide^1^, and its use has extended to other dementias^2^. The CDR^®^ provides two different measures: 1. the CDR^®^ Global Score uses a series of rules to categorize participants into 5 categories; 2. the CDR^®^ Sum of Boxes (CDR^®^-SB) in which the score is the sum of all the domain scores in the CDR^®^ and it can range from 0 to 18. Both measures are highly correlated but the CDR^®^-SB is preferred because it provides greater precision for tracking changes in patients with cognitive decline^3^. Consequently, the CDR^®^-SB has been used as a primary outcome in dementia research including randomized clinical trials and observational studies in AD^4^.

The CDR^®^-SB should reflect only the patient’s cognitive impairment stage and be free of bias related to non-disease factors. It is known that both biological characteristics (sex) as well as sociodemographic constructs (gender) can affect Alzheimer’s disease pathology, presentation and diagnosis^5^. However, less is known about the effects of sex and gender on reporting of symptoms. Previous research has shown that females and males enduring the same health condition report different dimensions and symptoms as the most relevant for their well-being ^6^, and females also tend to report a higher number of symptoms than males with the same diagnosis^7^. These differences in the dimensions and number of symptoms reported can result in a sex bias in which health personnel might diagnose more or less frequently females and males suffering from the same disease^8^. Gender plays a significant role in caregivers’ awareness and likelihood of reporting symptoms in patients with AD. Women often assume a primary caregiving role and are better at detecting and reporting subtle changes in cognition, behaviour, and functioning in their partners. This heightened perceptiveness contributes to early detection for AD^9^. Studies indicate that female caregivers are more inclined to notice and report symptoms of depression^10–12^, a potential risk factor and prodromal symptom for dementia. Moreover, gender stereotypes may inadvertently influence the evaluation scales used for assessing AD, impacting the interpretation of caregiver reports.

Furthermore, in the next of kin of patients with cognitive decline, the perception of caregiving and its burden is different between both sexes, with women experiencing more mood issues and expressing more openly their concerns, while men are less willing to acknowledge their level of distress to not appear weak or vulnerable^13^. Nevertheless, both sexes experience a high burden associated with the experience. Also, additional information on caregivers of patients with cognitive decline has shown that these differences in caregiving burden are not limited to sex differences but also to relationship with the patient as children experience a higher burden of care than the spouses^14^. However, little is known about how the informant’s characteristics affect the CDR^®^-SB score in patients with AD.

We think that these gender and sex differences in informant’s perception and verbalization may impact the reporting of information about the persons under their care, and thus affect the staging score of patients with cognitive impairment. We hypothesize that informant characteristics are associated with differences in CDR^®^-SB scores. Our aim was to assess whether informant characteristics, such as sex, relationship to the patient, frequency of contact, and their potential interactions were associated with varying CDR^®^-SB scores in patients diagnosed with AD diagnosis at the mild cognitive impairment (MCI) or dementia stage included in the National Alzheimer’s Coordinating Center Uniform Data Set (NACC-UDS).

## Methods

We included participants from the NACC-UDS, which is an ongoing longitudinal prospective study that started in 2005 and is collecting standardized data of participants with varying cognitive statuses, ranging from normal cognition to MCI and persons living with dementia. The NACC-UDS includes all the data from the Alzheimer’s Disease Research Centers, funded by the National Institute on Aging (USA)^15^. Longitudinal studies are a design in which the same patients are seen more than one time, and in each evaluation, the same data is collected in a cross-sectional manner. We included patients with a diagnosis of MCI or dementia due to presumptive etiologic diagnoses of AD who had a Mini-Mental State Examination (MMSE) or Montreal Cognitive Assessment (MoCA) score and with informant information regarding sex and relationship to the patient. We included all the NACC-UDS visits available that fulfill the inclusion criteria.

Our outcome was the CDR^®^-SB score, and from the NACC-UDS information we evaluated the following informant characteristics: a) sex as a dichotomous variable; b) relationship to the patient as: Spouse or Partner, Child, or other relationship; and c) how often the informant contacted the patient in the following categories: 1) less than once a week; 2) at least once a week 3) daily; and 4) lives with the patient. Regarding patients’ information, we included z standardized MMSE or MoCA scores as a covariable to control for the patient’s cognitive state; when both measurements were available, we selected the MMSE scores. Also, we included the patient sex as we wanted to explore the interactions of patient sex with informant sex, informant sex with relationship, and patient sex with relationship.

To analyze longitudinal data, we require a model that can estimate the outcome in every visit as the lower level of information, but accounting for the fact that the data is from the same patient which becomes the clustering factor; also, the model should estimate how much of the variation is because the data came from the same patients. To fulfill these requirements, we performed conditional growth models using a multilevel linear regression model with random intercepts to account for the longitudinal structure of the data. The first level of analysis was every visit, and the second level grouping variable was the NACC-UDS participant. Also, we included the time since the first visit to account for the potential effect of time on the CDR^®^-SB scores, and we allowed a random slope for time.

First, we performed a model with all the proposed interactions, then we eliminated those interactions that were non-significant for the model one at the time, and we compared with the previous model using an ANOVA; finally, we used an ANOVA to compare our final model with the model without any interaction to ensure that the interaction was significant for the model. The final model includes only the significant interaction of participant sex and the relationship between participant and informant. We report in accordance with STROBE reporting guidelines.

## Results

We included data from 20636 participants with patients’ mean age at the initial visit 74.0 ± 9.4 years and 11157 (54.1%) were females. The full patient demographics from their first visit are presented in Table 1. We included a total of 47727 visits with a median of 2 (1–4) visits per patient, the informants had a mean age of 66.2 ± 13.2 years, were female in 32987 (69.1%) visits, and 28878 (60.5%) were spouse or partner to the patients, 12745 (26.7%) were the patients’ children and 6104 (12.8%) had other relationship to the patients. Regarding the frequency of informant contact with the patient, 4006(8.4%) visited less than once a week, 8653 (18.1%) visited at least once a week, 2470 (5.2%) visited daily and 32598 (68.3%) informants lived with the patient.

Table 2 presents the results from our full model. We found that the CDR^®^-SB scores were higher by 0.20 (CI 95%: 0.11 to 0.29) if the informant was a female. When comparing with informants who were spouses or partners, the CDR^®^-SB scores were 0.39 (CI 95%: 0.25 to 0.53) higher if the informant was a child, and if the relationship was other, the CDR^®^-SB scores were lower by 0.18 (CI 95%: −0.35 to −0.01).

We observed that the patient’s sex had a non-significant p value for the model meaning that CDR^®^ scores were not different due to patients being female or male. Nonetheless, we retained patient sex in our model due to the observation that the interaction between relationship and patient sex when the informant was other than spouse or child and the patient was female resulted in a CDR^®^-SB score 0.24 (CI95%: 0.03 to 0.46) higher than the effect of relationship category of other in male patients. Regarding the frequency of contact between informant and patient, using less than weekly visit as a reference, the CDR^®^-SB scores were 0.38 higher (CI95%: 0.28 to 0.47) when the informant visited the patient at least once a week, the CDR^®^-SB was 0.65 higher (CI95%: 0.52 to 0.78) in the case of informants that visited the patient daily, and the CDR^®^-SB score was 0.57 (CI95%: 0.46 to 0.69) higher for the informants living with the patients. As expected, an increase of 1 SD on the z-standardized MMSE or MoCA is associated with lower CDR^®^-SB scores by −3.27 (CI 95%: −3.3 to −3.25).

Finally, the random component of our model showed that the Intraclass Correlation Coefficient (ICC) was 0.62, which can be interpreted as that 62% of all the variance in the model is because the data was collected from the same patients and not due to the changes between one follow up and the others, even after including the cognitive scores at every visit.

In the supplementary file, we present the ANOVA results when comparing the full interaction model with the final model and the comparison of the final model with a model without interactions (Table S1).

## Discussion

The CDR^®^-SB is a scale that was designed to reflect a patient’s stage of disease by collecting information from a patient’s informant ^1^. However, our analysis found that the CDR^®^-SB scores were also affected by informant characteristics and the relationship between patients and informants. Interestingly, we found that females as informants tend to report higher scores (by 0.20 points) as compared to male informants; we also found that the CDR^®^-SB scores were 0.39 points higher if the informant was a child of the patient. Importantly, we found that the CDR^®^-SB scores are higher with increased frequency of contact between the informant and the patient. Our findings are relevant because the CDR^®^-SB has become one of the preferred outcomes in AD clinical trials, including the recently approved AD disease-modifying therapy Lecanemab where the difference between the intervention and placebo arms in CDR^®^-SB was −0.45 (CI95%: −0.67 to −0.23)^16^, a difference of similar magnitude as our findings of the effect of informants on the scores.

As previously stated, the CDR^®^ is a scale designed to reflect the cognitive stage of patients with dementia, and our results with an ICC of 0.62 confirm that most of the score variation is due to the patient’s characteristics. However, even after controlling for the patient characteristics, the informant sex, the relationship to the patient and frequency of contact are still significantly impacting the CDR^®^-SB scores.

To the best of our knowledge, this is the first report on the impact of informant characteristics on the CDR^®^-SB. Unraveling the underlying reasons behind our results is likely intricate and multifactorial. For instance, female caregivers of patients with dementia experience a higher prevalence of depression and emotional involvement than male caregivers^13^, potentially influencing their responses. This higher prevalence of depression may contribute to an increased reporting of symptoms in the care recipient, as previous research has shown that individuals with depressive disorders tend to harbour a more negative perception about themselves and significant others^17^. Moreover, women, who are typically more engaged in personal care, invest more caregiving hours for individuals with disabilities^18^; this may result in women being able to collect more information about the patients and the extent of their limitations. Another factor to consider is that spouses and adult children may experience the caregiving of patients with dementia differently. A US study revealed that adult children caregivers experienced a higher burden of care, and reported a lower quality of life compared to spousal caregivers, despite children reporting better social supports^14^. This heightened perception of caregiving burden might manifest in a increased perception of the patient’s limitations. Lastly, more frequent interaction between the informant and the patient are expected to enhance knowledge about the patient’s limitations in accomplishing common tasks, including those that are not performed daily. However, informants with more frequent interactions or those living with the patients may also experience a higher caregiving burden ^19^, potentially leading to an amplified perception of the patient’s dependence.

Our study has several strengths, firstly, we used information from a large dataset with standardized information that increases precision and reduces the risk of measurement bias. Secondly, by restricting to AD patients we have selected the population for which the CDR^®^ instrument was designed and has been validated with the highest reproducibility. Thirdly, we included in the model the z-standardized MMSE or MoCA score which allowed us to control for a potentially large source of confound and increased the accuracy of the informant characteristics’ effect on the CDR^®^-SB. Finally, performing a multilevel model allowed us to use all the encounters available and at the same time account for the inter-patient variability making the estimation more precise.

However, we acknowledge some limitations in our study. Firstly, the NACC-UDS records the patient and informant’s sex, but it does not report the patient’s gender. Some part of these differences might be due to cultural gender differences in the social roles, and not related to the biological sex; at the moment we cannot distinguish this stratified effect. This lack of information on the effect of sex and gender in dementia research is a pervasive problem; a recent scoping review shows that most studies do not distinguish between the two concepts and tend to aggregate them as a single concept or use the two concepts interchangeably^20^.

Also, we lack information about the CDR^®^ raters training to perform the CDR^®^-SB and how they are prepared to understand and translate the information that the informant is providing. Physicians using informant information might interpret the report of symptoms differently if the informant is male or female. Previous research has shown that in psychiatrists measuring their encounter perception with the Assessment of Clinician’s Subjective Experience instruments, there are higher degrees of tension and difficulty in tuning to male patients than to female patients^21^ which results in greater difficulty in being empathetic and understanding towards the other person’s experience. This factor may extend to the healthcare interaction between informants and health providers and impact the CDR^®^ and it should be further evaluated in future research. Finally, due to the observational nature of the longitudinal study, it is possible that the result might be a consequence of other unmeasured variables.

The CDR^®^-SB is one of the most commonly used scales to stage dementia and to measure disease evolution in patients with dementia. Our findings suggest that the CDR^®^-SB is, however, subject to the influence of the informants’ characteristics, hence something that is not related to the patient’s cognitive status. Given our results, the effect of informant characteristics on all informant-based scales should be assessed and possibly factored into the calculation. Furthermore, our results argue for the use of concomitant measures, objective biomarkers, and updating measurement scales norming acknowledging informants’ sex and characteristics.

In summary, our study revealed that the CDR^®^-SB score among tNACC-UDS patients with an AD diagnosis were influenced by informant characteristics, including sex, relationship to the patient, frequency of contact between the patient and informant, and the interaction between patient sex and the informant’s relationship. It is crucial to address and comprehend these associations as the CDR^®^-SB should ideally remain robust to such factors, and be staging only the AD in the patients. In conclusion, the significance of our findings is underscored by the widespread use of the CDR^®^-SB as a primary endpoint in AD clinical trials. This prompts a reassessment of the appropriateness of exclusively relying solely on the CDR^®^-SB as the sole outcome measure in AD research. Future research should investigate the influence of additional informant variables on the CDR^®^-SB and explore methodologies to control or adjust the CDR^®^-SB scores, accounting for the influence of these informant-related characteristics.

## Figures and Tables

**Table 1:** Description of included NACC-UDS patients.

Variable		N = 20636
Sex (%)	Female	11157 (54.1)
	Male	9479 (45.9)
Age at initial visit (mean ± SD)		74.0 ± 9.4
Race (%)	White (Non-Hispanic)	15702 (76.1)
	African American (Non-Hispanic)	2601 (12.6)
	Hispanic	1711 (8.3)
	Other	622 (3.0)
Primary Language (%)	English	18916 (91.7)
	Spanish	1255 (6.1)
	Other	465 (2.3)
Years of education (mean ± SD)		15.1 ± 6.2
Marital status (%)	Married or living with a partner	13667 (66.2)
	Widowed	4076 (19.8)
	Divorced or separated	2045 (9.9)
	Never married	765 (3.7)
	Other or unknown	83 (0.4)
Living situation (%)	Lives alone	2602 (16.5)
	Lives with spouse or partner	10351 (65.6)
	Lives with relative or friend	1806 (11.5)
	Lives with group	578 (3.7)
	Other	408 (2.6)
	Unknown	27 (0.2)
Cognitive status (%)	MCI	7856 (38.1)
	Dementia	12780 (61.9)
CDR^®^-SB (mean ± SD)		4.3 ± 3.9

NACC-UDS: National Alzheimer’s Coordinating Center Uniform Data Set. CDR^®^-SB: The Clinical Dementia Rating^®^ Sum of Boxes
